# Safety and Feasibility of Primary Closure Following Laparoscopic Common Bile Duct Exploration for Treatment of Choledocholithiasis

**DOI:** 10.1007/s00268-022-06871-9

**Published:** 2022-12-29

**Authors:** Lunjian Xiang, Jingjing Li, Dingzhi Liu, Lang Yan, Hongrui Zeng, Yan Liu

**Affiliations:** 1 Department of Hepatobiliary Surgery, Chongqing University Three Gorges Hospital, 165 Xincheng Road, Wanzhou District, Chongqing, China; 2 Department of Ultrasound, Chongqing University Three Gorges Hospital, 165 Xincheng Road, Wanzhou District, Chongqing, China

## Abstract

**Background:**

T-tube drainage following laparoscopic common bile duct (CBD) exploration may lead to T-tube displacement and water–electrolyte disorders, affecting patients’ quality of life. In particular, biliary peritonitis may develop in a small number of patients after T-tube removal, requiring reoperation. This prospective cohort study was performed to investigate the safety and feasibility of primary closure following laparoscopic CBD exploration for the treatment of choledocholithiasis.

**Methods:**

Patients who were treated for choledocholithiasis by laparoscopic CBD exploration with primary closure from January 2019 to March 2022 comprised the PC group (*n* = 145). Patients who were treated for choledocholithiasis by laparoscopic CBD exploration with T-tube drainage during this period comprised the TD group (*n* = 153). Perioperative and follow-up outcomes were collected and statistically analyzed.

**Results:**

The TD and PC groups showed significant differences in the operation time (124.6 ± 40.8 vs. 106 ± 36.4 min, *P* = 0.000) and postoperative hospital stay (7.1 ± 2.6 vs. 5.9 ± 2.0 days, *P* = 0.000). No significant difference was observed in terms of blood loss, the ratio of conversion to laparotomy, and postoperative parameters. Preoperative albumin and total bilirubin levels were the risk factors of bile leakage after surgery. No patients developed CBD stricture or carcinogenesis, The rates of residual and recurrent stones in the TD and PC groups were 1.97% vs. 1.40% and 1.31% vs. 1.40%, respectively, with no significant difference (*P* = 1.000 for both).

**Conclusions:**

Primary closure following laparoscopic CBD exploration is safe and feasible for selected patients with choledocholithiasis.

## Introduction

Choledocholithiasis has an incidence of 1–15% and is present in 5–29% of patients with cholelithiasis [1]. Choledocholithiasis can lead to obstructive jaundice, biliary pancreatitis, and even acute obstructive suppurative cholangitis, which may be life-threatening; thus, timely and effective surgical treatment is required [2, 3]. The conventional treatment for choledocholithiasis is T-tube drainage following open common bile duct (CBD) exploration. In recent decades, laparoscopy and choledochoscopy have become increasingly popular, and laparoscopic suturing and knotting techniques are being constantly improved. T-tube drainage following laparoscopic CBD exploration has therefore become an essential treatment for choledocholithiasis with advantages such as a small wound, rapid postoperative recovery, and a high success rate [4–7].

Despite its advantages, an indwelling T-tube may lead to T-tube displacement, water–electrolyte disorders, and extension of the postoperative recovery time, all of which affect patients’ quality of life. In particular, biliary peritonitis develops in a small number of patients after T-tube removal and requires reoperation. The complication rate of indwelling T-tubes can reach 10.5–20.0% [8–11]. Primary closure following laparoscopic CBD exploration can avoid the limitations of an indwelling T-tube; however, the problems of bile leakage, residual CBD stones, and stricture still require clinicians’ attention. Therefore, this treatment remains controversial. Retrospective studies have shown that primary closure following laparoscopic CBD exploration can shorten the hospital stay and reduce the development of postoperative complications [12, 13].

On this background, we performed a prospective cohort study to investigate the safety and feasibility of primary closure following laparoscopic CBD exploration for the treatment of choledocholithiasis.

## Materials and methods

### Participants and grouping

This study included all consecutive patients with choledocholithiasis who met the inclusion criteria and were hospitalized in the Department of Hepatobiliary Surgery, Chongqing University Three Gorges Hospital from January 2019 to March 2022. The TD group comprised patients treated by T-tube drainage following laparoscopic CBD exploration, and the PC group comprised patients treated by primary closure following laparoscopic CBD exploration.

### Inclusion and exclusion criteria

The same inclusion and exclusion criteria were used for both groups. The inclusion criteria were: (1) a definitive preoperative diagnosis of choledocholithiasis; (2) a CBD diameter of ≥ 8 mm; and (3) a favorable general condition, good function of vital organs, and tolerance of general anesthesia. The exclusion criteria were: (1) preoperative complications such as acute obstructive suppurative cholangitis or gallbladder perforation requiring emergency surgery; (2) severe upper abdominal adhesion hindering insertion of endoscopic surgical devices or the establishment of carbon dioxide pneumoperitoneum; (3) a tumor in the biliary tract or stricture at the lower portion of the CBD as revealed by intraoperative choledochoscopy, necessitating a change in the surgical approach; and (4) an internal fistula of the biliary tract and digestive tract as detected by intraoperative exploration.

### Surgical procedure

General anesthesia with endotracheal intubation was used for both groups of patients, and all operations were performed by specialists. The patient was placed supine in the dorsal elevated position, and the operating table was tilted to the left by 15–30°. CO_2_ pneumoperitoneum was established below the umbilicus, and the pneumoperitoneum pressure was maintained at 11–13 mmHg (1 mmHg = 0.133 kPa). Trocars were placed using a four-port technique. Using electrocautery to anatomy, the cystic duct and cystic artery which were clamped with ligation clips (Teleflex Medical, Rancho El Descanso, Tecate, Mexico) and then, cutoff. After anterograde excision, the gallbladder was removed through the main operating port. Next, the anterior wall of the CBD was longitudinally incised at the upper portion of the duodenum using electrocautery and followed by insertion of a choledochoscope (Olympus Corp., Shinjuku, Tokyo, Japan) into the CBD through the main operating port. Stones were removed using a stone extractor (Cook Medical, Bloomington, IN, USA), and the biliary tract was flushed with normal saline and repeatedly inspected with the choledochoscope.

In the PC group, 4–0 or 5–0 absorbable sutures (Ethicon, Inc., Somerville, NJ, USA) were used to close the CBD using an intermittent everting suture technique. In the TD group, a silicone T-tube of appropriate size (16–22 Fr) was inserted into the CBD, followed by closure using the same suture technique. The abdominal cavity was repeatedly flushed with normal saline, and the operation was completed after inserting a drainage tube through the foramen of Winslow. If no postoperative bile leakage was observed, the abdominal drainage tube was removed 48–72 h postoperatively. At 6 weeks postoperatively, patients in the TD group underwent T-tube angiography and then, removed.

### Data collection

The following data were collected for both groups of patients.

- Preoperative parameters: sex, age, comorbidities, American Society of Anesthesiologists (ASA) class, white blood cell (WBC) count, liver function indices, electrolytes, CBD diameter, CBD stones (single or multiple), state of the gallbladder, and concomitant acute pancreatitis
- Intraoperative parameters: operation time, blood loss, and ratio of and reason for conversion to laparotomy
- Postoperative parameters: hospital stay, anal exhaust time, WBC count, liver function indices, electrolytes, and complications
- Follow-up parameters: residual CBD stones, stone recurrence, stricture, and carcinogenesis

### Definitions

- Bile leakage: Determined according to the definition and grading of severity by the International Study Group of Liver Surgery [14]
- ASA class: Determined according to the ASA Physical Status Classification [15]
- Operation time: Duration from skin incision to incision closure
- Postoperative hospital stay: Duration from the day of the operation to hospital discharge
- Discharge criteria: The abdominal drainage tube was removed in both group; the T-tube was closed in the TD group; the patient’s mental, dietary, and sleep conditions are favorable; and the patient’s activity level is normal

### Re-examination and follow-up

At 6 weeks postoperatively, T-tube angiography was performed in the TD group to check for residual biliary stones, biliary stricture, and the T-tube location. Hepatobiliary ultrasonography was performed in the PC group by a single sonographer and ultrasound device (Analogic Corp., Peabody, MA, USA) to check for residual biliary stones and biliary stricture; if stones were suspected, magnetic resonance cholangiopancreatography (MRCP) was carried out for further diagnosis. Both groups of patients then underwent abdominal ultrasonography by a single sonographer every 6 months to check for recurrence of biliary stones, biliary stricture, and carcinogenesis; if imaging revealed suspected stones, stricture, or carcinogenesis, MRCP or enhanced computed tomography was performed. Researchers from the Department of Hepatobiliary Surgery, Chongqing University Three Gorges Hospital followed up the patients by telephone and outpatient visits.

### Statistical analysis

Data analyses were performed using the IBM SPSS Statistics for Windows, Version 24.0 (IBM Corp., Armonk, NY, USA). Measurement data are presented as mean ± standard deviation and were analyzed using the t test or Wilcoxon’s rank sum test, whereas count data were analyzed using the *χ*2 test or Fisher’ exact test. Factors that influenced bile leakage were analyzed by logistic regression.

## Results

### Preoperative parameters

Statistical analyses revealed no significant differences in age, sex, ASA class, comorbidities, CBD diameter, number of CBD stones, status of the gallbladder, WBC count, liver function, electrolytes, or number of patients with concomitant acute pancreatitis between the TD and PC groups (Table 1).

**Table 1:** General information of patients treated by T-tube drainage (TD group) and primary closure (PC group) following laparoscopic common bile duct exploration

Parameters	TD group	PC group	*P*
	(*n* = 153)	(*n* = 145)	
Sex (male/female)	62/91	55/90	0.261
Age (year)	60.9 ± 15.8	59.4 ± 16.1	0.919
*Comorbidity*			
Hypertension	23	19	0.261
T2DM	6	5	0.439
Hypertension and T2DM	7	5	0.172
Coronary heart disease	1	1	0.962
ASA (I/II/III)	28/57/68	24/51/70	0.306
WBC (× 109)	6.8 ± 2.8	6.6 ± 3.1	0.652
*Preoperative electrolytes*			
K + (mmol/L)	4.0 ± 0.4	4.0 ± 0.3	0.801
Na + (mmol/L)	139.7 ± 3.6	139.7 ± 3.0	0.923
Cl-(mmol/L)	104.1 ± 3.7	104.3 ± 3.2	0.485
*Preoperative liver functions*			
Albumin (g/L)	39.1 ± 5.0	39.2 ± 5.1	0.713
Total Bilirubin (μmol/L)	52.3 ± 72.4	40.0 ± 52.0	0.679
Direct Bilirubin (μmol/L)	36.7 ± 48.2	31.3 ± 45.8	0.316
ALT (U/L)	130.0 ± 122.3	128.7 ± 135.5	0.930
AST (U/L)	89.9 ± 94.6	86.8 ± 108.2	0.789
Diameter of CBD (mm)	11.2 ± 2.8	11.3 ± 2.7	0.672
No. of CBD stones (single/multiple)	74/79	71/74	0.368
*State of the gallbladder*			
Gallstone	111	104	0.261
Gallbladder removed	23	23	0.368
No. of cases with acute pancreatitis	12	11	0.136

Data are presented as n or mean ± standard deviation

*T2DM*, type 2 diabetes mellitus; *ASA*, American Society of Anesthesiologists; *WBC*, white blood cell; *ALT*, alanine aminotransferase; *AST*, aspartate aminotransferase; *CBD*, common bile duct

### Perioperative outcomes

The TD and PC groups showed significant differences in the operation time (124.6 ± 40.8 vs. 106 ± 36.4 min, *P* = 0.000) and postoperative hospital stay (7.1 ± 2.6 vs. 5.9 ± 2.0 days, *P* = 0.000). However, no significant between group differences was observed in blood loss (56.2 ± 54.5 vs. 53.8 ± 50.6 mL, *P* = 0.692), the ratio of conversion to laparotomy (2.6% vs. 2.1%, *P* = 1.000), the anal exhaust time (2.2 ± 0.7 vs. 2.1 ± 0.7 days, *P* = 0.096), or the postoperative WBC count, liver function, and electrolytes. In the TD group, three patients developed postoperative complications (bile leakage in two, pulmonary infection in one). In the PC group, four patients developed postoperative complications (biliary leakage in three, incomplete intestinal obstruction in one). The postoperative complication rate was not significantly different between the two groups (2.0% vs. 2.7%, *P* = 0.717) (Tables 2 and 3). The logistic regression analysis showed that the preoperative albumin and total bilirubin concentrations were independent risk factors for bile leakage (Table 4).

**Table 2:** Intraoperative parameters of patients treated by T-tube drainage (TD group) and primary closure (PC group) following laparoscopic common bile duct exploration

Parameters	TD group	PC group	*P*
	(*n* = 153)	(*n* = 145)	
Operation time (min)	124.6 ± 40.8	106 ± 36.4	0.000
Blood loss (ml)	56.2 ± 54.5	53.8 ± 50.6	0.692
Ratio of conversion to laparotomy (%)	2.6	2.1	1.000
Severe gallbladder inflammation and poor presentation of the gallbladder triangle	4	3	

Data are presented as mean ± standard deviation or n unless otherwise indicated

**Table 3:** Postoperative outcomes of patients treated by T-tube drainage (TD group) and primary closure (PC group) following laparoscopic common bile duct exploration

Parameters	TD group	PC group	*P*
	(*n* = 153)	(*n* = 145)	
Postoperative hospital stay (days)	7.1 ± 2.6	5.9 ± 2.0	0.000
Postoperative anal exhaust time (days)	2.2 ± 0.7	2.1 ± 37.7	0.096
Postoperative complication rate (%)	2.0	2.7	0.717
Bile leakage	2	3	
Pulmonary infection	1	0	
Incomplete ileus	0	1	
WBC (× 109)	12.7 ± 3.5	12.1 ± 4.0	0.125
*Postoperative electrolytes*			
K + (mmol/L)	4.06 ± 0.39	4.09 ± 0.41	0.465
Na + (mmol/L)	137.7 ± 3.5	138.7 ± 2.8	0.056
Cl-(mmol/L)	103.5 ± 3.5	104.3 ± 3.5	0.090
*Postoperative liver functions*			
Albumin (g/L)	34.2 ± 4.5	34.7 ± 4.5	0.289
Total bilirubin (μmol/L)	29.9 ± 34.1	30.4 ± 38.4	0.914
Direct bilirubin (μmol/L)	21.7 ± 28.6	21.7 ± 33.2	0.997
ALT (U/L)	92.4 ± 71.3	93.3 ± 73.6	0.916
AST (U/L)	63.3 ± 46.3	59.8 ± 37.7	0.485

Data are presented as mean ± standard deviation or *n*

*WBC*, white blood cell; *ALT*, alanine aminotransferase; *AST*, aspartate aminotransferase

**Table 4:** Risk factor analysis of bile leakage after surgery

	*P*	OR	95% confidence interval	
			Upper limit	Lower limit
Surgical approach (primary closure vs T-tube drainage)	0.090	7.602	0.729	79.286
Sex (male vs female)	0.379	2.257	0.369	13.813
Age	0.263	0.996	0.908	1.027
WBC	0.975	0.994	0.679	1.456
Albumin	0.025	0.790	0.642	0.971
Total bilirubin	0.012	0.979	0.648	0.912
Direct bilirubin	0.560	1.014	0.968	1.061
ALT	0.519	0.998	0.991	1.005
AST	0.782	0.999	0.990	1.007
Diameter of CBD	0.359	0.850	0.609	1.203
No. of CBD stones (single/multiple)	0.121	6.574	0.609	70.939
Operation time	0.643	0.999	0.969	1.020
Blood loss	0.954	0.999	0.980	1.019

*OR*, odds ratio; *WBC*, white blood cell; *ALT*, alanine aminotransferase; *AST*, aspartate aminotransferase; *CBD*, common bile duct

### Follow-up outcomes

Patients in the TD group were followed up for 3–40 months (median, 20.0 months), and patients in the PC group were also followed up for 3–40 months (median, 17.5 months). The follow-up time was not significantly different between the two groups. During the follow-up period, four patients in the TD group developed T-tube prolapse from the CBD. One of them developed an internal fistula with the duodenum, and one developed biliary peritonitis after T-tube removal, which was treated by reoperation. One and two patients died in the TD and PC groups, respectively, and each death was attributed to other diseases. The mortality rate was not significantly different between the groups (0.65% vs. 1.37%, *P* = 1.000). No patients developed CBD stricture or carcinogenesis during follow-up. Nevertheless, residual stones were found in three and two patients in the TD and PC groups, respectively, with stone recurrence in two patients of each group. The rates of residual and recurrent stones in the TD and PC groups were 1.97% vs. 1.40% and 1.31% vs. 1.40%, respectively, with no significant difference (*P* = 1.000 for both) (Table 5).

**Table 5:** Follow-up outcomes of T-tube drainage (TD group) and primary closure (PC group) following laparoscopic common bile duct exploration

Parameters	TD group	PC group	*P*
	(*n* = 153)	(*n* = 145)	
Follow-up time (months)	20.0(3–40)	17.5(2–40)	0.695
Mortality (n, %)	1(0.65)	2(1.37)	1.000
Residual stone rate (n, %)	3(1.97)	2(1.40)	1.000
Stone recurrence rate (n, %)	2(1.31)	2(1.40)	1.000
Biliary stricture	0	0	–
Bile duct carcinogenesis	0	0	–

Data are presented as median (range), *n* (%), or *n*

## Discussion

The treatment of choledocholithiasis has evolved from conventional open surgery to minimally invasive surgery. Because of the large surgical wound, slow postoperative recovery, and long hospital stay, conventional open surgery has been gradually replaced by minimally invasive surgery. McCune et al. [16] reported the first endoscopic retrograde cholangiopancreatography (ERCP) procedure in 1968, which signified the emergence of ERCP-based endoscopic stone extraction as a minimally invasive surgical approach for the treatment of choledocholithiasis. However, ERCP-based stone extraction must be integrated with endoscopic sphincterotomy of the duodenal papilla. Consequently, patients are faced with risks such as complicated acute pancreatitis, hemorrhage, and duodenal perforation, as well as intestinal fluid reflux and stone recurrence caused by damage to the sphincter of Oddi. Hence, ERCP-based stone extraction should be selected with caution [17, 18]. With the popularization of laparoscopy and choledochoscopy plus the advancement of laparoscopic suturing and knotting techniques, T-tube drainage following laparoscopic CBD exploration has emerged as an essential treatment for choledocholithiasis. However, an indwelling T-tube may cause cholangitis, formation of an internal fistula with the digestive tract, and water–electrolyte disorders, affecting patients’ work and daily life. Furthermore, a small number of patients develop bile leakage-induced peritonitis after T-tube removal and thus, require reoperation. Although primary closure following laparoscopic CBD exploration can avoid the limitations of an indwelling T-tube, it is still associated with problems such as bile leakage, residual CBD stones, and stricture; therefore, it remains controversial [19–22].

We compared the perioperative and follow-up outcomes of T-tube drainage versus primary closure following laparoscopic CBD exploration through the present prospective cohort study. We found significant differences in the operation time and postoperative hospital stay, with better results in the PC than TD group. There were no evident between group differences in the intraoperative blood loss or ratio of conversion to laparotomy; postoperative anal exhaust time, complication rate, WBC count, liver function, or electrolytes; or rates of residual and recurrent stones during follow-up. Additionally, no patients in either group developed CBD stricture or carcinogenesis during follow-up. The results of this study indicate that primary closure following laparoscopic CBD exploration is safe and feasible for selected patients with choledocholithiasis.

Bile leakage is a common short-term complication after bile duct exploration. Among the 298 patients included in this study, five (1.7%) developed bile leakage (three in the PC group, two in the TD group). This incidence is lower than that found in a previous study (11.3%) [23], which might be attributed to our selection of patients and accumulation of suturing and surgical skills. All cases of bile leakage spontaneously resolved after unobstructed drainage. Using logistic regression, we identified the preoperative albumin and total bilirubin concentrations as independent risk factors for bile leakage, irrespective of an indwelling T-tube. Indeed, research has shown that an indwelling T-tube can be an alternate option if other procedures failed and cannot prevent bile leakage [24, 25]. In the present study, the CBD approach was used for stone extraction in all cases. For extraction of CBD stones through the cystic duct approach, patients with a thick cystic duct and stones at the lower portion of the CBD are often selected to facilitate choledochoscope insertion and stone extraction [26].

According to previous studies, the rate of T-tube-related complications, including bile leakage after T-tube removal, ranges from 10.5% to 20.0% [8–11]. In the present study, four patients in the TD group developed T-tube prolapse from the CBD during follow-up; one of them developed an internal fistula with the duodenum, and one developed biliary peritonitis after T-tube removal requiring reoperation. The operation time and hospital stay were shorter in the PC than TD group, consistent with previous reports [11, 12, 27].

Residual CBD stones are another short-term complication after CBD exploration. In the present study, three (1.97%) and two (1.40%) patients had residual stones in the TD and PC groups, respectively, with no significant difference between the two groups. The residual stone rate of 1.40% in the PC group was lower than that in previous research [28], and this inconsistency may be associated with our repeated full course examination of the intrahepatic and extrahepatic bile ducts using intraoperative choledochoscopy. We determined the presence of residual stones at the cutoff time of 6 weeks postoperatively. In the TD group, residual CBD stones were detected in three patients by T-tube angiography and were successfully removed through a T-tube sinus tract. In the PC group, residual stones were detected in two patients through abdominal ultrasonography and were successfully removed by laparoscopic CBD exploration with primary closure.

CBD stone recurrence, stricture, and carcinogenesis are long-term complications after CBD exploration and essential indicators of the safety and feasibility of primary closure. During follow-up, two patients each in the TD and PC groups developed stone recurrence. The stone recurrence rates in the two groups were 1.31% and 1.40%, respectively, which were not significantly different. The recurrent stones were successfully removed by laparoscopic CBD exploration with primary closure in all four patients. Despite the absence of CBD stricture and bile duct carcinogenesis throughout the follow-up period, further observation and follow-up are still needed because of the relatively short follow-up time. Primary closure following laparoscopic CBD exploration can be used as the treatment strategy for patients with choledocholithiasis and those who have previously undergone gallbladder or CBD surgery. In the present study, 23 patients in the PC group had previously undergone cholecystectomy. The safety and feasibility of the treatment have also been demonstrated in a previous study [29].

## Conclusion

Primary closure following laparoscopic CBD exploration complies with the characteristics of bile metabolism in the human body. It avoids the disadvantages of an indwelling T-tube while shortening the operation time and postoperative hospital stay. Additionally, there are no significant differences in the rates of residual and recurrent CBD stones compared with those resulting from an indwelling T-tube. Accordingly, primary closure following laparoscopic CBD exploration is safe and feasible for selected patients with choledocholithiasis. However, the safety and feasibility of this treatment require further verification by large sample, multicenter, prospective randomized case–control studies.
