# Correction: Safety and Feasibility of Primary Closure Following Laparoscopic Common Bile Duct Exploration for Treatment of Choledocholithiasis

**DOI:** 10.1007/s00268-023-06905-w

**Published:** 2023-01-19

**Authors:** Lunjian Xiang, Jingjing Li, Dingzhi Liu, Lang Yan, Hongrui Zeng, Yan Liu

**Affiliations:** 1 Department of Hepatobiliary Surgery, Chongqing University Three Gorges Hospital, 165 Xincheng Road, Wanzhou District, Chongqing, China; 2 Department of Ultrasound, Chongqing University Three Gorges Hospital, 165 Xincheng Road, Wanzhou District, Chongqing, China

**Correction to: World Journal of Surgery** https://doi.org/10.1007/s00268-022-06871-9

In the original online version of this article, A. Al Samaraee's name in Ref. 7 was misspelled.

The original article was corrected.

